# Computational models of the Posner simple and choice reaction time tasks

**DOI:** 10.3389/fncom.2015.00081

**Published:** 2015-07-02

**Authors:** Carolina Feher da Silva, Marcus V. C. Baldo

**Affiliations:** ^1^Department of General Physics, Institute of Physics, University of São PauloSão Paulo, Brazil; ^2^Department of Physiology and Biophysics, Institute of Biomedical Sciences, University of São PauloSão Paulo, Brazil

**Keywords:** reaction time, attention, Posner task, Bayesian model, neural network

## Abstract

The landmark experiments by Posner in the late 1970s have shown that reaction time (RT) is faster when the stimulus appears in an expected location, as indicated by a cue; since then, the so-called Posner task has been considered a “gold standard” test of spatial attention. It is thus fundamental to understand the neural mechanisms involved in performing it. To this end, we have developed a Bayesian detection system and small integrate-and-fire neural networks, which modeled sensory and motor circuits, respectively, and optimized them to perform the Posner task under different cue type proportions and noise levels. In doing so, main findings of experimental research on RT were replicated: the relative frequency effect, suboptimal RTs and significant error rates due to noise and invalid cues, slower RT for choice RT tasks than for simple RT tasks, fastest RTs for valid cues and slowest RTs for invalid cues. Analysis of the optimized systems revealed that the employed mechanisms were consistent with related findings in neurophysiology. Our models predict that (1) the results of a Posner task may be affected by the relative frequency of valid and neutral trials, (2) in simple RT tasks, input from multiple locations are added together to compose a stronger signal, and (3) the cue affects motor circuits more strongly in choice RT tasks than in simple RT tasks. In discussing the computational demands of the Posner task, attention has often been described as a filter that protects the nervous system, whose capacity is limited, from information overload. Our models, however, reveal that the main problems that must be overcome to perform the Posner task effectively are distinguishing signal from external noise and selecting the appropriate response in the presence of internal noise.

## 1. Introduction

In the last decades, scientific interest in visual attention has grown, with many studies focusing on the behavioral effects of attention (Carrasco, 2011). One such effect—faster reaction times (RTs)—has been extensively investigated for more than a century (Schmidgen, 2002) and, since the landmark experiments by Posner in the late 1970s (Posner, 1980), considered one of the key behavioral consequences of attention, along with enhanced detection.

**Figure 1:**
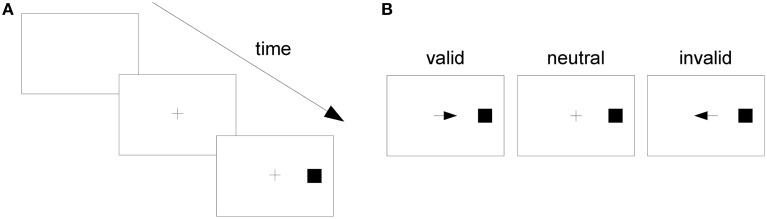
**The Posner task**. **(A)** A trial in the Posner task. **(B)** From left to right, a valid, a neutral, and an invalid cue.

In one of Posner's experiments, subjects fixed their gaze upon the center of a screen (Figure 1A). Then, in a central location near the fixation point, a cue was presented, which could be an arrow pointing to the left or right side of the screen or a plus sign. Subjects were instructed to pay attention to the side of the screen pointed to by the cue or to divide their attention between both sides if the cue was a plus sign. After a varying time interval had elapsed, a stimulus (the square in Figure 1A) appeared on one side of the screen. This was the target stimulus, to which the subject was instructed to respond as fast as possible by pressing a key. If the subject mistakenly responded before target onset, this was considered an “anticipated response;” if the subject missed the target and did not respond within a specified time window after target onset, this was considered a “slow response.” In both cases, the response was considered an error and subjects were notified of it.

If the target appeared on the side pointed to by the cue, the cue was considered “valid;” if it appeared on the opposite side, it was considered “invalid.” If the cue was a plus sign, providing no information on the side the target would appear, it was considered “neutral” (Figure 1B). When the cue pointed to a side, the probability that the target would appear on the indicated side was 0.8 and on the opposite side, 0.2. When the cue was neutral, the target might appear on either side with probability 0.5. Posner analyzed RTs separately for different cue types (valid, neutral, and invalid) and observed that RT was shortest when the cue was valid and longest when it was invalid. He attributed such differences to the effect of voluntary attention and proposed that attention speeds up the processing of stimuli presented at its focus. His task has since become known as the “Posner task,” and it has been considered a “gold standard” test of spatial attention (Coull et al., 2011).

The present study aims to contribute to the understanding of attention by analyzing the performance of the Posner task—what its computational demands are and what neural mechanisms underlie its performance. We have developed two computational models and optimized them to perform the Posner task: a sensory Bayesian model, which performs noisy signal detection, and a motor neural network model, which performs action selection. By developing two separate models for the sensory and the motor components, we were able to examine separately the role of attention in perceptual processing and in action selection. Our models were able to replicate the main experimental features of RT experiments with human subjects, such as the relative frequency effect, suboptimal RTs and significant error rates due to noise and invalid cues, faster RTs for valid cues and slower RTs for invalid cues, and slower RT for choice reaction time (CRT) tasks, wherein subjects must select an appropriate response to the target, than for simple reaction time (SRT) tasks, wherein there is only one possible response to all targets. More importantly, our results have enabled us to approach and discuss two long-standing issues in attention research.

The first issue concerns the role of selective attention in perception and action. The standard view on selective attention is that the nervous system can only deal with a limited amount of sensory information (Broadbent, 1958); when there is a stimulus overload, selection mechanisms are activated to ensure the processing of high priority stimuli (Desimone and Duncan, 1995). Posner himself discussed his results from this perspective (Posner, 1980), and it is still widely accepted today—in a 2011 review of visual attention research, attention has been defined as “a selective process, which is usually conceptualized as being related to limited cognitive and brain resources” (Carrasco, 2011). Nevertheless, evidence indicates that attention might be necessary regardless of limited capacity. Noise has been shown to affect the results of RT tasks (Eriksen and Hoffman, 1973; Dosher and Lu, 2000b; Lu et al., 2002), which suggests that attention is important for filtering out noise; also, attention might play a role in decision making and action selection (Allport, 1987; Dayan et al., 2000; Feher da Silva et al., 2008; Wu, 2011; Krauzlis et al., 2014). Our results support the two latter proposals. We have found that the main results of Posner's experiment can be understood in terms of noisy signal detection and action selection, with no need to assume limited capacity. Thus, our models contribute to a broader view of attention.

Another issue in attention research is that the mechanisms of visual attention are not well-understood. It has been much discussed whether attention enhances the signal, reduces noise or changes decision criteria (Carrasco, 2011). Our Bayesian model supports the latter view. More specifically, we propose that attention has a multiplicative effect on stimuli that can be identified with the prior probabilities that the target will appear at a given location (spatial attention) or at a given time (temporal attention). A higher prior probability assigned to the location indicated by the cue can explain the so-called “attentional effect”—the difference between RT for invalid cues and RT for valid cues. Additionally, our neural network model suggests that in CRT tasks, part of the attentional effect is motor, due to an activation of the ipsilateral motor circuit and an inhibition of the contralateral motor circuit by the cue. Finally, the Posner task may also be affected by the different frequencies assigned to different cue types. We propose other mechanisms involved in the Posner task—(1) in SRT tasks, input from multiple locations are added together to compose a stronger signal, and (2) in CRT tasks, the cue affects motor circuits much more strongly than in SRT tasks—as well as provide evidence for previously proposed mechanisms, such as the competition between potential actions through mutual inhibition and a threshold for perceptual decision making.

Our two computational experiments are described below. In experiment 1, a Bayesian system is built to detect a target in a noisy environment. The signal-to-noise ratio is varied, as well as accuracy levels, leading to a speed-accuracy trade-off. In experiment 2, a neural network model is used to explore motor control and action selection in the Posner task by changing the frequency of cue types and the level of internal noise.

## 2. Experiments

The code for these experiments can be downloaded from https://github.com/carolfs/rtexp and is licensed under the GNU General Public License v3.0.

### 2.1. Experiment 1—sensory model

The purpose of Experiment 1 was to study noisy signal detection in the Posner task. To that end, a Bayesian detection system was built to perform target detection in a RT task with varying signal-to-noise levels. This model is perceptual only, and RT corresponds to the time the target has been detected. This has allowed us to study noisy signal detection separately from action control.

The task starts with a 100-time-unit window wherein the target will be presented at a random time unit on one of two sides, left or right. At each time unit, the system calculates the probability that the target has already appeared. When this probability reaches a preset value γ, for instance, 0.9, the system responds. It calculates separately the probability that the target has already appeared on the left side and the probability that the target has already appeared on the right side. The probability that the target has already appeared regardless of its location is the sum of those two probabilities. In a SRT task, the system considers only the probability that the target has already appeared regardless of side in order to decide when to respond. In a CRT task, it considers the probability for each side separately, so it can try to respond correctly. At the beginning of the task, the probabilities that the target has already appeared on either side are zero. At each time unit, the system updates them based on two new stimuli, one from the left side and one from the right side, each of which consists of the sum of normally distributed noise (μ = 0, σ = 2) and, if the target is present at that location, a signal of preset intensity. The target appears on one of the sides at a time unit randomly selected with uniform distribution from the 100 time units, then remains at that location until the task is over.

Since the system is optimal, modeling a subject that has already learned to perform the task, it takes all the available information into account, never missing the target in SRT tasks, since the probability that the target has already appeared in the 100th time unit is 1.0—it is an RT task without catch trials. It also assigns different prior probabilities to each location depending on a previously provided cue, which may point to the left side, to the right side, or be neutral. When the cue pointed to a side, the probability that the target would appear on the indicated side was 0.8 and on the opposite side, 0.2; when the cue was neutral, the target might appear on either side with probability 0.5.

#### 2.1.1. Methods

A Bayesian detection model was built to detect a target in a RT task. On each trial, a target appears at time *t*, 1 ≤ *t* ≤ *t*_*max*_ = 100, and at one of two possible locations, the *left* side or the *right* side, both time and location randomly selected with uniform distribution. The Bayesian model calculates, at every instant, the probability that the target has already appeared on the left and the probability that the target has already appeared on the right. In a SRT task, it responds when the probability that the target has already appeared, which is the sum of those two probabilities, reaches a preset value, γ, 0 ≤ γ ≤ 1. In a CRT task, it responds when any of the two probabilities reaches γ. After the target appears, it stays at the same location until the end of the trial. In a SRT task, the system always responds before *t*_*max*_, because the probability that the target has already appeared is 1 at *t*_*max*_. In a CRT task, if the system does not respond before *t* = 1000, the response is considered slow. Also, in a CRT task, the system responds left or right depending on which probability has reached γ. If both probabilities reach γ at the same time, the system responds left or right with probability 0.5.

Let *p*(*T*_*l*_(*t*)) be the probability that the target has appeared on the left exactly at time *t*, *p*(*T*_*r*_(*t*)) the probability that the target has appeared on the right exactly at time *t*, and *p*(*T*(*t*)) the probability that the target has not appeared yet at time *t*. Then *p*(*T*(*t*)), the probability that the target has appeared (on any side) exactly at time *t* is *p*(*T*(*t*)) = *p*(*T*_*l*_(*t*)) + *p*(*T*_*r*_(*t*)). Also, *p*(*T*(*t*)) = 1/*t*_*max*_ if 1 ≤ *t* ≤ *t*_*max*_ and 0 otherwise, and *p*(*T*(*t*)) + *p*(*T*(*t*)) = 1. The values of *p*(*T*_*l*_(*t*)) and *p*(*T*_*r*_(*t*)) depend on the information relayed by the cue. If, for instance, the cue points to the left, then the target will appear on the left with probability 0.8; therefore, *p*(*T*_*l*_(*t*)) = 0.8/*t*_*max*_ and *p*(*T*_*r*_(*t*)) = 0.2/*t*_*max*_. Generally, let ρ be the probability that the target will be presented on the left, as indicated by the cue. Then *p*(*T*_*l*_(*t*)) = ρ/*t*_*max*_ and *p*(*T*_*r*_(*t*)) = (1−ρ)/*t*_*max*_.

The probability that the target has *already* appeared at time *t*′, i.e., it has appeared at time *t*′ or at any earlier time, is *p*(*T*(*t* ≤ *t*′)) = *t*′/*t*_*max*_. Likewise, *p*(*T*_*l*_(*t* ≤ *t*′)) and *p*(*T*_*r*_(*t* ≤ *t*′)) can be obtained by multiplying *p*(*T*(*t* ≤ *t*′)) by ρ or (1 − ρ), respectively, and *p*(*T*(*t* ≤ *t*′)) = 1 − *p*(*T*(*t* ≤ *t*′)), which is the probability that the target has not appeared yet at time *t*′.

In order to detect the target, the system also considers a pair of stimuli *S*(*t*) = (*S*_*l*_(*t*), *S*_*r*_(*t*)) at each time *t*, one from the left and one from the right. Each stimulus is a randomly selected number with normal distribution. The standard deviation of the distribution, σ, is the intensity of the noise. The mean of the distribution is zero when the target is not on that side and *s*, the intensity of the signal, otherwise. Thus, a stimulus can have a negative value, but this has no physical significance: a negative stimulus is just a stimulus whose intensity is below the mean when the target is absent.

Each pair of stimulus *S*(*t*) will be assigned a likelihood depending on whether it is assumed that the target has already appeared on the left or on the right or the target has not appeared yet:

(1) $$f(S(t^{\prime })|T_{l}(t\leq t^{\prime }))=\exp\left(\frac{-{\left(S_{l}(t^{\prime })-s\right)}^{2}}{2\sigma^{2}}\right)\exp\left(\frac{-{\left(S_{r}(t^{\prime })-0\right)}^{2}}{2\sigma^{2}}\right)$$

(2) $$f(S(t^{\prime })|T_{r}(t\leq t^{\prime }))=\exp\left(\frac{-{\left(S_{l}(t^{\prime })-0\right)}^{2}}{2\sigma^{2}}\right)\exp\left(\frac{-{\left(S_{r}(t^{\prime })-s\right)}^{2}}{2\sigma^{2}}\right)$$

(3) $$f(S(t^{\prime })|\bar{T}(t\leq t^{\prime }))=\exp\left(\frac{-{\left(S_{l}(t^{\prime })-0\right)}^{2}}{2\sigma^{2}}\right)\exp\left(\frac{-{\left(S_{r}(t^{\prime })-0\right)}^{2}}{2\sigma^{2}}\right)$$

The likelihood functions are joint functions for two independent random variables: the stimuli on the right and on the left are independent if it is assumed that the target has already appeared on a given side or it has not appeared yet. They are based on the probability density function of the normal distribution, ignoring the proportionality constant.

If the target appeared on the left at time *t*_*t*_, it is possible to calculate the likelihood of a sequence of stimuli *SS*(*t*′) = (*S*(1), *S*(2), …, *S*(*t*′)):

(4) $$f(SS(t^{\prime })|T_{l}(t_{t}))=\prod_{i\;=\;1}^{n}f_{i}(S(i))$$

where

(5) $$f_{i}(S(i))=\{\begin{array}{ll} f(S(i)|\bar{T}(t\leq i)), & \text{if }i<t_{t};\\ f(S(i)|T_{l}(t\leq i)), & \text{otherwise.} \end{array}$$

Similarly, the likelihood of the sequence can be calculated if the target appeared on the right at time *t*_*t*_. If the target has not appeared yet, the likelihood of the sequence is simply:

(6) $$f(SS(t^{\prime })|\bar{T}(t\leq t_{t}))=\prod_{i=1}^{n}f(S(i)|\bar{T}(t\leq i))$$

At each time *t*' of the trial, the Bayesian system calculates the probability that the target has already appeared on the left and on the right, given the sequence of previous stimuli *SS*(*t*′) = (*S*(1), *S*(2), …, *S*(*t*′)). In a SRT task, it also calculates the sum of the two probabilities. It is:

(7) $$p(T(t\leq t^{\prime })|SS(t^{\prime }))=p(T_{l}(t\leq t^{\prime })|SS(t^{\prime }))+p(T_{r}(t\leq t^{\prime })|SS(t^{\prime }))$$

where

(8) $$p(T_{l}(t\leq t^{\prime })|SS(t^{\prime }))=\frac{g_{l}(t^{\prime })}{p(SS(t^{\prime }))}$$

(9) $$p(T_{r}(t\leq t^{\prime })|SS(t^{\prime }))=\frac{g_{r}(t^{\prime })}{p(SS(t^{\prime }))}$$

where

(10) $$p(SS(t^{\prime }))=g_{l}(t^{\prime })+g_{r}(t^{\prime })+g_{n}(t^{\prime })$$

(11) $$g_{l}(t^{\prime })=\sum_{t\;=\;1}^{t^{\prime }}f(SS(t^{\prime })|T_{l}(t))p(T_{l}(t))$$

(12) $$g_{r}(t^{\prime })=\sum_{t\;=\;1}^{t^{\prime }}f(SS(t^{\prime })|T_{r}(t))p(T_{r}(t))$$

(13) $$g_{n}(t^{\prime })=f(SS(t^{\prime })|\bar{T}(t\leq t^{\prime }))p(\bar{T}(t\leq t^{\prime }))$$

In a SRT, the model responds if *p*(*T*(*t* ≤ *t*′)|*SS*(*t*′)) ≥ γ. In a CRT task, it responds left if *p*(*T*_*l*_(*t* ≤ *t*′)|*SS*(*t*′)) ≥ γ or right if *p*(*T*_*r*_(*t* ≤ *t*′)|*SS*(*t*′)) ≥ γ. It is thus guaranteed that the number of anticipated responses will not exceed γ times the number of trials.

These formulas can be simplified into an iterative and more computationally efficient form (not shown here; see the Github project for the algorithm).

#### 2.1.2. Results

**Figure 2:**
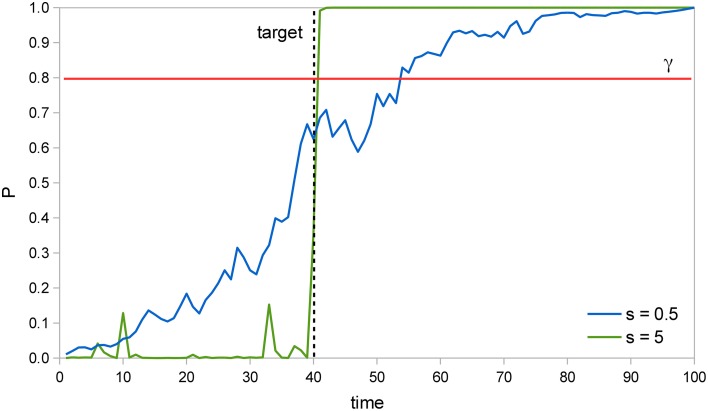
**Example trials in a SRT task**. In two example trials of a SRT task where the target is presented at *t* = 40 (dashed line), the probability that the target has already appeared *P* increases much faster after the target is presented when signal intensity *s* is 5 than when it is 0.5. Thus, *P* reaches the perceptual threshold γ = 0.8 (red line) earlier in the former situation, which results in a faster RT.

Figure 2 illustrates how the Bayesian system is able to detect the target faster as the signal intensity *s* increases. In two example trials of a SRT task with a valid cue, the probability that the target has already appeared *P* is calculated at each time instant and the system responds when *P* reaches the perceptual threshold value γ = 0.8. The target actually appears at *t* = 40; when *s* = 5, detection occurs soon afterwards, at *t* = 41, but when *s* = 0.5 it occurs much later, at *t* = 54. Moreover, we can see that when *s* = 5, *P* remains very low at first, then rises abruptly once the target appears, while when *s* = 0.5, *P* rises more uniformly throughout the trial. The former curve reflects the inpu*t*′s likelihood, which is very low before the target appears, but very high afterwards. The latter curve reflects instead the prior probability that the target has already appeared, which rises constantly.

**Figure 3:**
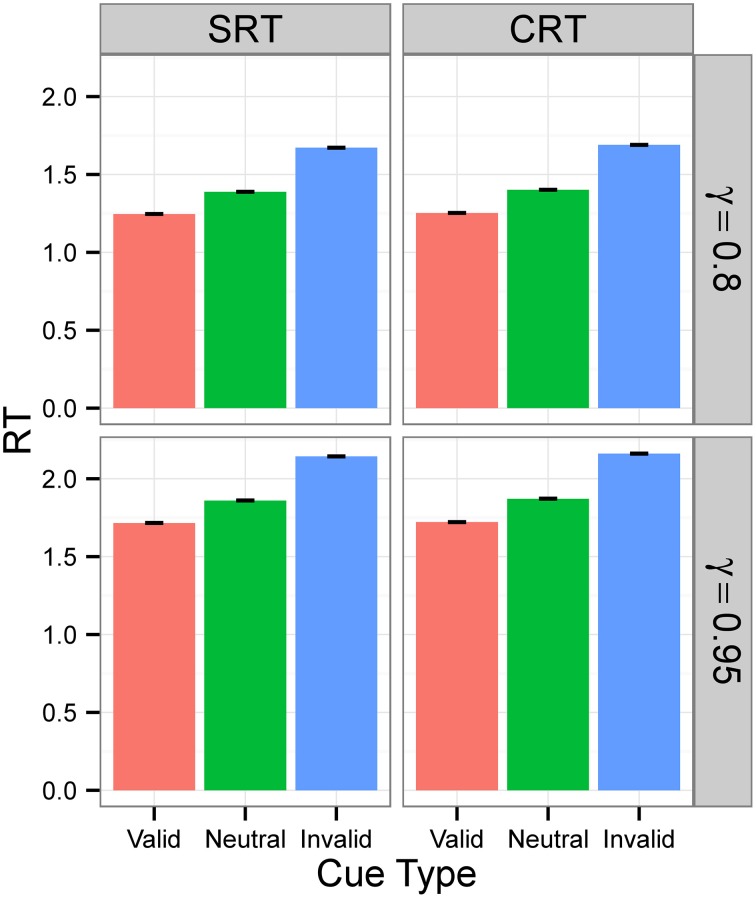
**RTs in Experiment 1 for signal intensity 5**. RTs for different tasks (SRT or CRT) and γ-values (0.8 or 0.95) when signal intensity is 5. The values are the average RTs over 10^6^ trials. RTs are given in the model's arbitrary time units.

**Figure 4:**
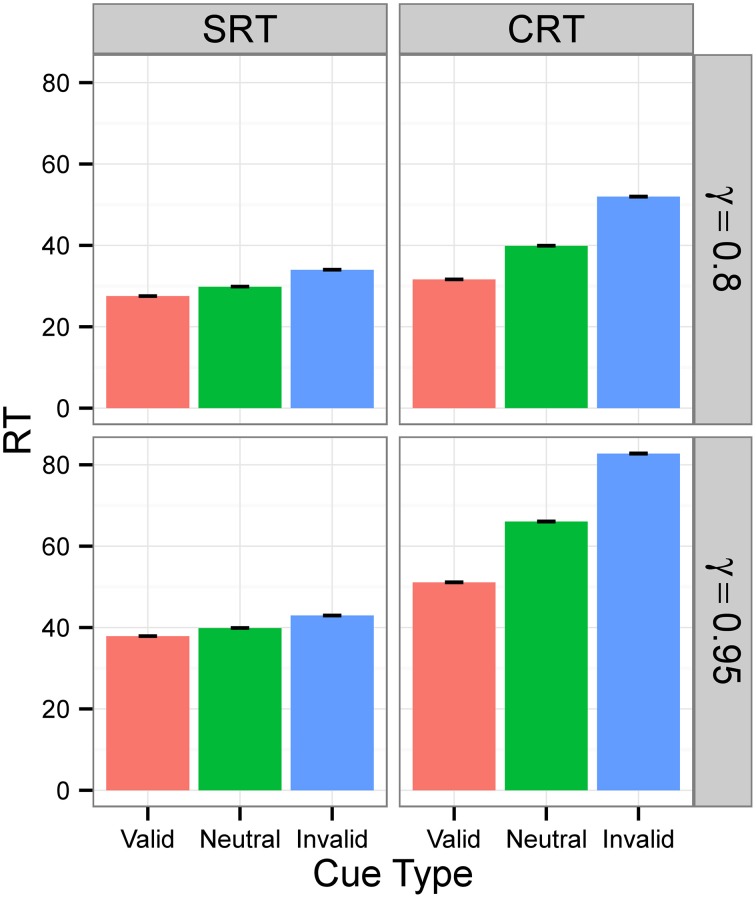
**RTs in Experiment 1 for signal intensity 0.5**. RTs for different tasks (SRT or CRT) and γ-values (0.8 or 0.95) when signal intensity is 0.5. The values are the average RTs over 10^6^ trials. RTs are given in the model's arbitrary time units.

Figures 3, 4 display the average RTs for different tasks (SRT or CRT), signal intensities (5 or 0.5), and γ-values (0.8 or 0.95). In every case, RT is shortest for the valid cue and longest for the invalid cue, both in SRT and in CRT tasks. Also, RTs are longer when γ is high and much longer when stimulus intensity is low. They are also longer for the CRT task than for the SRT task.

When signal intensity was high, accuracy rates—the proportion of correct trials—were also high, at 94% for γ = 0.8 and 99% for γ = 0.95. When signal intensity was low, accuracy varied according to the task. For the SRT task, accuracy rates were 82% for γ = 0.8 and 96% for γ = 0.95. For the CRT task, accuracy also varied according to the cue type, and are given for valid, neutral, and invalid cues, in this order: 88, 83, and 63% for γ = 0.8, and 98, 96, and 88% for γ = 0.95. The difference in accuracy between cue types was mostly due to the proportions of incorrect responses, which were 2, 10, and 27% for γ = 0.8 and 1, 3, and 11% for γ = 0.95. The proportion of slow responses was 0% for both tasks in all conditions. In SRT tasks, all errors were due to anticipated responses and in CRT tasks, they were due to both anticipated and incorrect responses.

The results have shown that the Bayesian model detects the target faster for valid cues than for neutral cues, both in SRT and in CRT tasks, as a result of using different priors for each cue type. In this respect, it reproduces the results obtained from experiments with humans. It also exhibits a clear speed-accuracy trade-off.

#### 2.1.3. Discussion

Experiment 1 illustrates the speed-accuracy trade-off observed in RT tasks—naturally it takes less time for the system to be at least 80% sure (γ = 0.8) that the target has already appeared than for it to be at least 95% sure (γ = 0.95); therefore, the system will be faster to respond at a lower γ level, but it will also make more errors. It is clear that RTs in CRT tasks will be longer than in SRT tasks, because in CRT tasks responses are based on the probabilities that the target has already appeared on each side separately and, in SRT tasks, they are based on the sum of these probabilities. Thus, for the same input sequence, any γ level will be reached at least as fast in a SRT task as in a CRT task. The Bayesian model allowed us to vary the signal-to-noise ratio while maintaining high accuracy. The results indicate that the 5:2 ratio makes for an easy detection task, but at the 0.5:2 ratio, the system could only detect the signal many time units after target onset.

Calculating the probability that the target has already appeared on a given side involves multiplication by the prior probability that the target would appear on that side—0.8, 0.5 or 0.2, depending on whether the cue points to that side, is neutral or points to the opposite side. Thus, for the same input sequence, if the cue points to the left, for instance, the probability that the target has already appeared on the left at a given instant will be greater than or equal to what it would be if the cue was neutral or pointed to the right, since it was multiplied by 0.8 instead of 0.5 or 0.2. As a result, in CRT tasks, RT will be shortest for the valid cue and longest for the invalid cue.

In SRT tasks, RT will on average follow the same pattern, but in individual trials, the sum of the two probabilities, which responses are based on, may be higher when the cue is invalid than when it is valid, if during an invalid trial the probability that the target has already appeared on the cued side rises because of noise. This explains why the difference in RT between cue types is larger in CRT tasks than in SRT tasks.

### 2.2. Experiment 2—motor model

The purpose of experiment 2 is to investigate action control in the Posner task.

Totally connected neural networks of integrate-and-fire neurons were built to perform SRT and CRT tasks and a genetic algorithm was used to find synaptic weights and biases for the networks. Genetic algorithms belong to the class of evolutionary algorithms, inspired by Darwin's theory of evolution, and can be seen as learning algorithms for neural networks, in which networks adapt through mutation, recombination, and selection. The experiment started with a set of randomly generated chromosomes, which determined the synaptic weights and biases of the neural networks, all floating-point numbers within an interval. The networks were then selected according to their performance in a RT task, so that the best networks would have a higher probability of generating offspring.

RT tasks were based on the experiments reported by Posner in 1980 (Posner, 1980). When the cue indicated a side, the target would appear on that side with probability 0.8 and on the opposite side with probability 0.2. When the cue was neutral, the target might appear on either side with probability 0.5.

**Figure 5:**
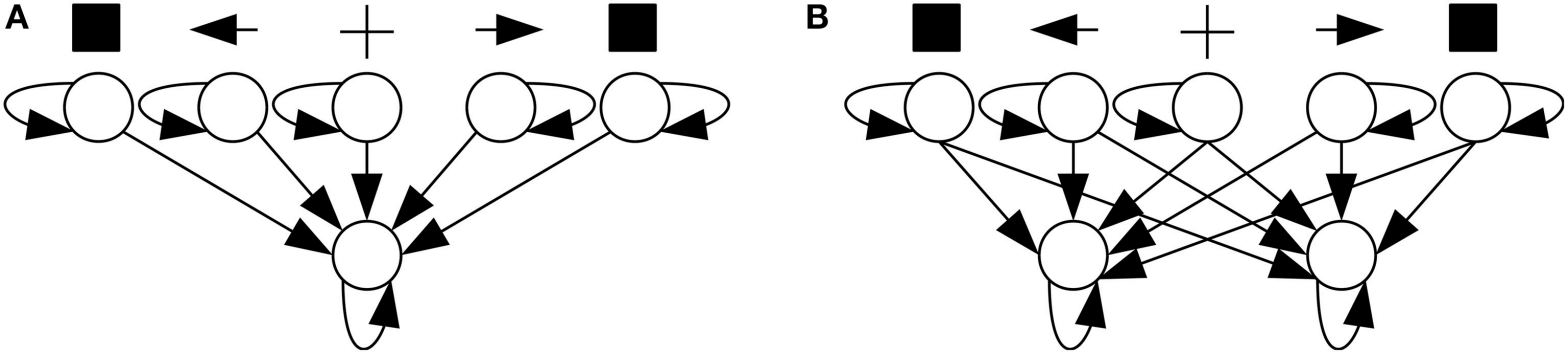
**Neural network architectures used in SRT (A) and CRT (B) tasks**.

In SRT tasks, neural networks had the architecture shown in Figure 5A^1^. A trial started with a reset of membrane potentials to their rest level, followed by 50 time units with no stimulation. Then a cue—a current added to one of the neural network's three central input neurons—was presented for a random interval of 100–200 time units. The cue could be neutral or point to one of the sides depending on the stimulated neuron. Afterward, a target stimulus—a current added to one of the two peripheral input neurons—was presented. Both the cue and the target stimuli had an intensity of 5 mV. When the output neuron fired, the neural network was considered to have responded to the target. The time elapsed between target onset and response was that trial's RT, and fast responses increased the network's performance and the chance that it would generate offspring. A response before target onset was considered anticipated and a response after 1000 time units after target onset was considered slow; such responses did not increase the network's performance.

In CRT tasks, neural networks had the architecture shown in Figure 5B. Trials proceeded identically to a SRT trial, except that a network was considered to have responded to the target when any of its two output neurons fired. If the target was presented on the left/right, the network had to respond with left/right output neuron. Incorrect responses, produced by the opposite output neuron, as well as anticipated and slow ones, did not increase performance.

RT tasks were performed in three different conditions of varying cue type proportions and levels of internal noise:

1. Condition A (Noise σ = 0, Cue 8:5:2): The proportion between valid, neutral, and invalid cues was 8:5:2, as used by Posner (1980), and noise was absent.
2. Condition B (Noise σ = 0, Cue 8:8:2): The proportion between valid, neutral, and invalid cues was set to 8:8:2, and noise was absent.
3. Condition C (Noise σ = 2, Cue 8:8:2): Every neuron was stimulated at every time unit with normally distributed noise (μ = 0, σ = 2) and a proportion of 8:8:2 between cue types was used.

Cue type proportions were altered from 8:5:2 to 8:8:2 because, if in Condition A RTs were shorter for valid cues than for neutral cues, such a result could be caused solely by the difference in relative stimulus frequencies. The usual proportion of 8:5:2 between cue types means that each possible input configuration, considering the locations of the cue and the target, has a different frequency in the RT task. For instance, trials with the cue pointing to the left and the target on the left are more frequent than trials with a neutral cue and the target on the left. If a neural network is able to respond faster when the target appears on the side pointed to by the cue, it will perform better than one that responds faster when the cue is neutral, just because one situation is more frequent than the other; as a consequence, within a few generations, the entire population is likely to exhibit the former behavior. By setting the proportion to 8:8:2 in Conditions B and C, trials with valid cues became just as frequent as trials with neutral cues, so any observed difference between RTs for valid cues and for neutral cues were not due to different stimulus frequencies. (In contrast, stimulus frequencies do not affect the results of Experiment 1, because the relative frequencies of valid and neutral cues are irrelevant to the task of detecting the target.)

Conditions A and B were performed in the absence of either external or internal noise, which is quite unrealistic; also, as previously mentioned, noise has been experimentally shown to affect the results of RT tasks. In order to assess the impact of internal noise on the present model, Condition C was designed.

#### 2.2.1. Methods

The neuron model in our experiments was the leaky integrate-and-fire model. It represents neurons by their membrane potential, which evolves according to the equation below:

(14) $$V(t+1)=V_{rest}+(V(t)-V_{rest})e^{-\frac{t}{\tau }}+I(t,b,w,\sigma ),$$

wherein *V*_*rest*_ is the neuron's rest potential, τ is the membrane's time constant, a positive real number, and *I* is the neuron's input at time unit *t*. *I* includes a bias, an external current, synaptic currents, and internal noise, and is constant during the considered time interval. An input coming from a synapse has the intensity of the synaptic weight when the pre-synaptic neuron fires an action potential. Thus, *I* is also a function of the network's parameters—its biases **b** and synaptic weights **w**—and noise intensity σ.

When the membrane potential reaches a threshold *V*_*threshold*_, it is considered that an action potential has occurred, without having its shape modeled, and the membrane potential is immediately restored to a value *V*_*reset*_. In our simulations, the following parameters were used: *V*_*rest*_ = *V*_*reset*_ = − 65 mV; V_*threshold*_ = − 40 mV; τ = 10 time units.

In a RT task, each trial proceeded according to the following description:

1. The neurons were reset to their initial condition: the membrane potential to its rest value and its input to the bias current.
2. The network didn't receive any stimulus for 50 time units.
3. A cue was presented, which might be neutral or indicate the left or the right side, for a time interval of uniform random duration between 100 and 200 time units.
4. A target was presented on the left or the side side, in addition to the cue.
5. The trial terminated when an output neuron fired or 1000 time units had elapsed after target onset.

Presenting the cue or the target meant stimulating the corresponding input neuron with an input current of 5 mV. The network's reaction time (*rt*) was calculated by subtracting the instant of response from the instant of target presentation. It was negative when the network responded before target presentation and positive otherwise. For the trials a correct response was emitted, the network was assigned a non-negative fitness value:

(15) $$f(rt)=\{\begin{array}{ll} 0, & \text{if }rt<0;\\ 1000e^{-0.01rt}, & \text{otherwise.} \end{array}$$

Thus, anticipated, wrong, and slow responses were awarded no fitness points. A network's total fitness value in a RT task was the sum of each trial's fitness value.

In Conditions A, B, and C, the number of trials in a task depended on the ratio between the number of neutral, valid, and invalid cues, and the target was presented both on the left and on the right side the same number of times for every cue type. Thus, the total number of trials was the sum of the numbers in a given cue ratio times two. Thus, when the ratio was 8:5:2, the task consisted of 30 trials and when the ratio was 8:8:2, the task consisted of 36 trials.

The networks' parameters (bias currents **b** and synaptic weights **w**) were optimized using a genetic algorithm. A chromosome was a set of genes representing the parameters as real numbers in the interval [−3, 3). The initial genes at generation 0 were randomly generated from a uniform distribution. One hundred chromosomes were generated in this manner and divided into five populations of 20 individuals. Neural networks were constructed based on these chromosomes and the networks performed the RT task. The fitness value was calculated for every network and used to select the parents of the next generation's individuals.

The fittest chromosome of each population was copied without change to the next generation of that population. Then, selection, crossover, and mutation were used to generate other 19 children for that population. Selection occurred by tournament—two chromosomes were selected randomly within a population and the winner became a parent. Child chromosomes inherited their parents' genes by crossover—for each gene, a parent was randomly selected and its gene was copied to the child chromosome—and mutation—with 5% probability, a number from the interval [−0.3, 0.3) was randomly generated with uniform distribution and added to the gene, but always keeping the gene in the interval [−3, 3). The populations evolved for 300 generations and at each 10 generations the fittest chromosome from each of the five populations migrated to a randomly chosen population, always keeping the number of individuals in each population at 20.

In order to calculate the mean RT, its standard deviation and its 95% confidence interval for a generation, the median RT for each cue type was calculated for each individual in a population, discarding slow, anticipated, and wrong responses. If an individual never responded correctly for a cue type, its median RT was considered null for that cue type. Mean and standard deviation values were calculated over the individual median RT values, discarding all null values.

#### 2.2.2. Results

**Figure 6:**
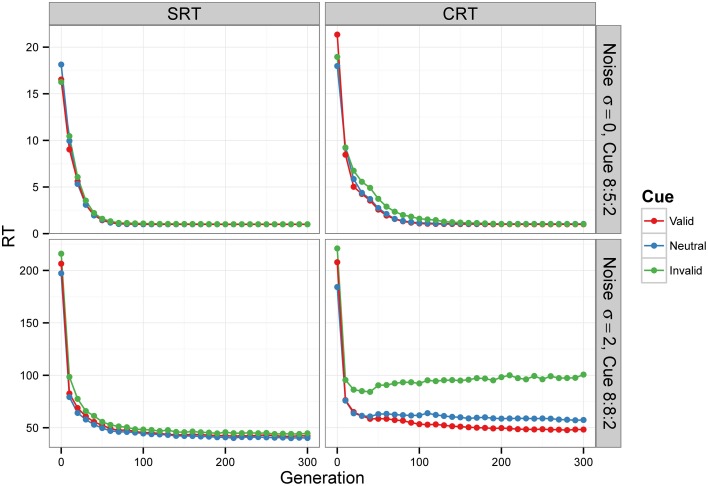
**Median RT evolution in Experiment 2—Conditions A and C**. The evolution of median RT for the valid, neutral, and invalid cues for SRT and CRT tasks and Conditions A (top) and C (bottom). Condition B is not shown—results were nearly identical to those of Condition A, because the noise level was also zero. RTs are given in the model's arbitrary time units.

**Figure 7:**
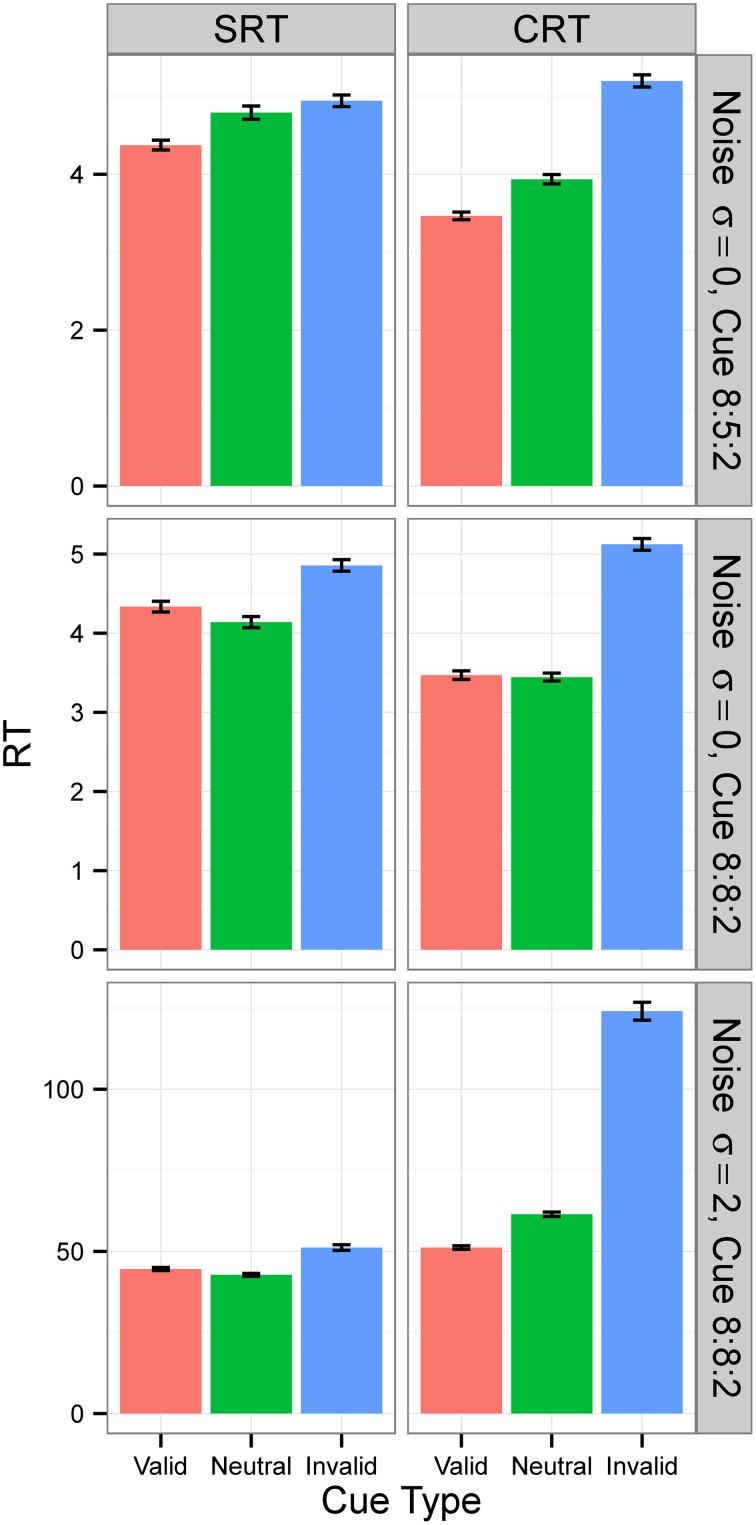
**Median RTs in Experiment 2**. Median RTs for SRT and CRT tasks. In Conditions A and B (top two rows), results were from generation 30 for SRT tasks and from generation 50 for CRT tasks. In Condition C (bottom row), all results are from generation 300. *N* = 50. RTs are given in the model's arbitrary time units.

For Condition A, the results, displayed in the top row of Figure 6, show that RTs decreased as the populations of neural networks evolved. By generation 300, RT was 1 time unit for all cue types. This is the minimum RT in correct trials, since the simulation advances in discrete time steps. At generation 0, the error rate (the sum of anticipated, slow, and incorrect response rates) was 100%, but from generation 30 onward, for the SRT experiment, and from generation 80 onward, for the CRT task, error rates were 0%. Thus, the neural networks achieved optimal performance, responding to the target at the earliest possible instant without errors. The top row of Figure 7 is a snapshot of the results before optimal performance was achieved, taken from generation 30 for SRT tasks and from generation 50 for CRT tasks. RTs were around 2–3 time units and error rates were below 2.5%. At that early point, RTs were shortest for valid cues and longest for invalid cues (the difference is statistically significant for all comparisons except those including the neutral cue in the SRT task, at the level of α = 0.05, with *N* = 50). The difference between RTs for valid and neutral cues might have been, however, due to stimulus frequency, as discussed above.

In Condition B, the proportion between valid, neutral, and invalid cues was 8:8:2, which made trials with neutral cues just as frequent as trials with valid cues. The middle row of Figure 7 is again a snapshot of the results before optimal performance was achieved, taken from generation 30 for SRT tasks and from generation 50 for CRT tasks. In the CRT experiment, average RTs for the valid and the neutral cue are both 2.63 time units, and in the SRT experiment, the RT for the neutral cue is shorter (3.0 time units) than the RT for the valid cue (3.2 time units). The difference is statistically significant for all comparisons except those including neutral cue in the CRT experiment, at the level of α = 0.05, with *N* = 50. These results confirm that the shorter RT for valid cues than for neutral cues found in Condition B was due to stimulus frequency. When valid cues were just as frequent as neutral cues, the RT for neutral cues was actually shorter than, or the same as, the RT for valid cues, depending on the task.

Condition C examines the effect of internal noise on RT. A proportion of 8:8:2 between cue types was used to decrease the effect of stimulus frequency. The bottom row of Figure 6 shows that although RT decreases as the populations of neural networks evolve, it never reaches the optimal value of 1. The bottom row of Figure 7 displays the results for generation 300. RTs are much longer than those obtained in previous conditions without noise. Also, they are longer for the CRT task than for the SRT task, similarly to what has been reported for humans and other animals. In the SRT experiment, RT for the neutral cue was shortest, but the difference between RTs for different cue types was small (42.06±0.98, 40.20±0.86, 44.68±1.32 time units for valid, neutral, and invalid cues, respectively, with *N* = 50). In the CRT experiment, RT for the valid cue was shortest and the difference between RTs for different cue types was large (48.27±1.40, 57.41±1.60, 100.68±6.63 time units for valid, neutral, and invalid cues, respectively, with *N* = 50). This indicates that, in CRT tasks, but not in SRT tasks, adding noise makes cue validity important apart from cue frequency. Error rates never dropped below 18% for the SRT experiment and 23% for the CRT experiment.

Examining the evolved parameters of the best-performing neural networks in Condition C, both for the SRT and for the CRT tasks, reveals that most of them vary widely. The ones that vary little (less than 5% of the total range of allowed values) are the positive bias of the output neurons (1.0) and the synaptic weights between the target neuron and the output neuron (in the SRT task) or the ipsilateral output neuron (in the CRT task), which is 2.9, close to the maximum. These features allowed networks to respond fast and correctly to the target.

**Figure 8:**
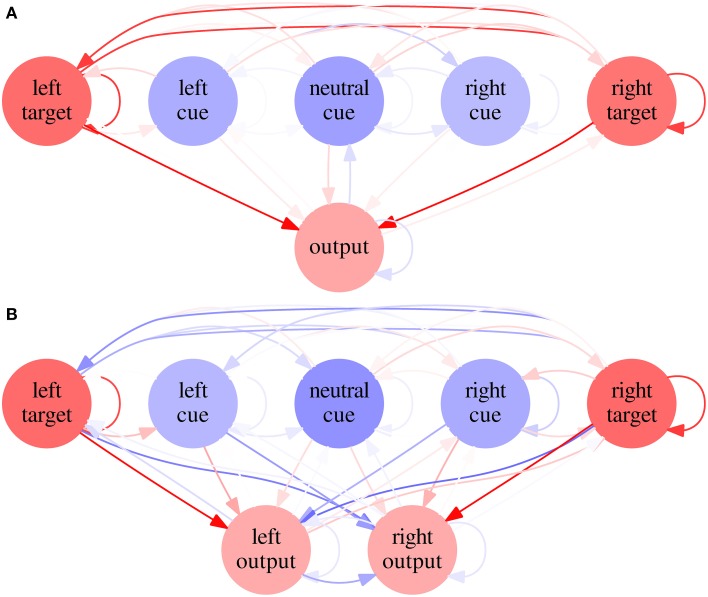
**Average networks in Experiment 2—Condition C**. Average network for SRT **(A)** and CRT **(B)** tasks in Condition C of Experiment 2. Excitatory biases and synapses are represented in red and inhibitory ones in blue, with proportional color intensity.

Average weights and biases are shown for every neuron and synapse in Figure 8. A neural network built from average parameters—an average network—does not necessarily represents well a population of networks. In this case, however, the average network's RTs are similar to the average RTs of the neural networks (39.13, 36.25, and 39.38 for the SRT and 48.14, 59.06, and 95.02 for the CRT, *N* = 1000). It is therefore useful to examine such networks in order to understand the results of Condition C. It is possible to see that, in the SRT average network, the target neurons are mutually excitatory. Tests indicate that when the target appears on one side, the ipsilateral target neuron fires under the target stimulus's direct stimulation, but the excitatory synapses between target neurons make the contralateral target neuron fire as well, albeit with a lower rate. The output neuron, under stimulation of both target neurons, fires faster than it would under stimulation of only one target neuron. In the CRT average network, the synapses between target and cue neurons and contralateral output neurons are inhibitory, which reduces the probability of incorrect responses, and the synapses between target and cue neurons and ipsilateral output neurons are excitatory, which is necessary for the network to respond correctly.

#### 2.2.3. Discussion

Condition A simply followed the basic procedure of the Posner task. The neural networks achieved optimal performance, responding to the target at the earliest possible instant, without errors. It shows how simple it is to build a machine that performs optimally such a simple task. Naturally, the machine is only able to perform the one task it was optimized for, and one wherein the signal and circuitry are free of noise.

The proportion 8:5:2 between cue types generated results whose overall pattern before complete optimization was the same as that of RT experiments with human subjects. Two explanations might have accounted for such results. Firstly, when the cue pointed to one side, the probability of the target appearing on that side was higher (0.8) than on the opposite side (0.2). When the cue was neutral, the target might have appeared on either side with equal probability. Thus, it was possible that RT was inversely proportional to the prior probability of the target location as indicated by the cue.

A second explanation is that RT was inversely proportional to input frequency. The proportion 8:5:2, in a Posner task, will result in the cue pointing to the left, to the right or being neutral with equal probability, that is, cue location frequencies are the same. But, when considering total input configuration, including cue and target locations, the left-cue-left-target configuration, for instance, constitutes 8/30 of trials, while the neutral-cue-left-target configuration constitutes 5/30 of trials and the right-cue-left-target configuration constitutes only 2/30 of trials. If a neural network is able to respond faster when the target appears on the same side as the cue, it will perform better than a neural network that responds faster when the cue and the target are on opposite sides, just because the target appears on the same side as the cue more frequently.

To test whether RT varied according to input frequency or to the probabilistic information provided by the cue, input frequencies were altered. Only the ratio between valid and neutral cues was altered, because altering the ratio between valid and invalid cues would also alter their probabilistic information. The proportion 8:8:2, used in Condition B, meant that valid configurations were just as frequent as neutral configurations; it also meant, however, that the neutral cue was more frequent than the left cue and the right cue. We observed that in CRT tasks, equaling the number of valid and neutral cues made the difference in RT between them disappear. SRT tasks, however, were affected by the larger number of neutral cues, as indicated by a slightly shorter RT for neutral cues than for valid cues in Conditions B and C.

Conditions A and B were not very realistic. In biological RT experiments, optimal performance is not achieved and both external (environmental) noise and internal (neural) noise disturb the information flow and generate uncertainty (Faisal et al., 2008). In an attempt to get a better model, internal noise was added to Condition C. Our results show that noise greatly increased RT, as well as the number of errors. Noise seems to be an essential element so that RT does not converge to the minimum value and might distribute itself over a wider range to reflect eventual modulation by other factors.

In the SRT task, we observed that target neurons activated each other and fired together. This mechanism amplifies the signal to the output neuron and explains why RT was shorter in the SRT task than in the CRT task. It works only because, in a SRT task, response is the same for all inputs. We also observed that RTs were similar for all cue types, with the shortest value for the neutral cue, which, as we have already seen, is more frequent than the right and the left cues. Even for the invalid cues, which were very infrequent, RT was not much longer than RT for other cue types, which indicates that different cue types had little effect on the output neuron. The same can also be inferred from the low-magnitude weights found for the synaptic weights between cue neurons and the other neurons.

In the CRT task, however, there was a sizable difference in RT between cue types. The lateral cue excited the ipsilateral output neuron and inhibited the contralateral output neuron, which made the RT for the valid cue shorter than the RT for the invalid cue. The probabilistic information provided by the cue was thus useful in the output stage, so that the correct output neuron was more likely to respond to the target than the incorrect one. At that stage, it was important to slow down the activation of output neurons so that they only fired after high, sustained sensory activity generated by the target, not after a momentary burst driven by noise.

## 3. General discussion

We have performed two experiments in order to understand the computational demands of the Posner task and propose mechanisms that allow its efficient performance. Experiment 1 uses a Bayesian model of noisy signal detection, which does not have a motor output or learn input frequencies; Experiment 2, on the other hand, uses a neural network model of action control, which does not distinguish signal from external noise. Using two different models for sensory and motor circuits has allowed us to study these two stages of neuronal processing separately.

### 3.1. Known mechanisms

Many of the mechanisms that were employed in our models have experimental support. In the Bayesian model, the gradual rise of a target probability that leads to a decision when it reaches a threshold is supported by results such as the recorded activity of neurons in the motor cortex of rhesus monkeys during RT tasks (Hanes and Schall, 1996; Roitman and Shadlen, 2002). This threshold mechanism has also been implemented as a neural network model of a perceptual CRT task without spatial cueing (Lo and Wang, 2006). More generally, it has been shown that Bayesian perceptual decision making can be implemented with probabilistic population codes (Ma et al., 2006; Drugowitsch and Pouget, 2012). The process of multiplying the two target probabilities by different priors is analogous to how attention is said to “bias” competition among neurons toward those that process stimuli from attended locations. Indeed, spatial attention has been found to exert a multiplicative effect on neurons in the V4 visual area, associated with object recognition (McAdams and Maunsell, 1999). Also, the prior probability that the target has already appeared, which increases during the time window within which the target may appear, can be identified with temporal attention. A study by Yu and Dayan (2004) shows that, in a complex discrimination task, a Bayesian network also exhibits shorter RT for valid-cue trials than invalid ones. Feldman and Friston (2010) also obtained similar results for a SRT task using a model that optimizes free-energy in a Bayesian fashion.

In the neural network model, the positive bias found for output neurons is analogous to motor preparation. In Condition C of Experiment 2, it had similar average values for SRT and CRT tasks, suggesting that motor preparation is independent of the task, which agrees with experimental results (Miller and Low, 2001). In CRT tasks, an output neuron was activated by the ipsilateral cue and target and inhibited by the contralateral cue, target, and output neuron. Inhibition between target and output neurons supports proposals of a mechanism of competition between potential actions through mutual inhibition (Cisek, 2007). Experimental evidence has been found of task-dependent response inhibition in the spinal cord following the target in uncued CRT tasks (Burle et al., 2004) and following an always valid, partially or fully informative cue in CRT tasks (Duque et al., 2010).

### 3.2. SRT vs. CRT tasks

In most biological and computational experiments, including all of ours, RTs are faster in SRT tasks than in CRT tasks. It is easy to see that the CRT is a more complex task, since it involves action selection, but how exactly does this complexity result in a slower RT?

With regard to the Bayesian system, it has already been explained that the system can detect that the target has already appeared before it can detect that the target has already appeared *on a particular side.* In neural networks, the output neuron is more strongly excited after target onset in the SRT task than in the CRT task. Although the cue neuron excites the ipsilateral output neuron more strongly in the CRT task than in the SRT task, the SRT network possesses excitatory synapses between both target neurons, so that both neurons fire when the target is presented. Such strategy can be generalized by noting that in a SRT task, after target onset, any excitatory activity incident on the output neuron is beneficial, because it decreases RT, even if the cause of such activity is, for instance, that the target was incorrectly detected on the opposite side. In a CRT task, however, an incorrect detection may cause the wrong output neuron to fire, leading to an incorrect response, so excitation of output neurons must be restricted, which leads to slower RTs.

Thus, both the Bayesian system and the neural networks display faster RTs for SRTs than for CRTs, because in SRTs the signal that leads to the response is the sum of input from multiple locations. To our knowledge, there is no experimental evidence for this proposal, which is an original contribution from our model.

### 3.3. The relative frequency effect

Conditions A and B of Experiment 2 demonstrate that stimulus frequency affects the result of RT tasks. The most frequent type of trial is the most important one, and optimizing for the most important case is a good strategy. In humans, the so-called “relative frequency effect” has been extensively studied in CRT tasks (e.g., Hyman, 1953; Sanders, 1970) and it was found that RT to more frequent stimuli tends to be faster than RT to less frequent stimuli. Relative stimulus frequency appears to affect both sensory and motor processes (Hawkins and Underhill, 1971; Dykes and Pascal, 1981). Similarly, RT to more frequent stimuli was also found to be faster than RT to less frequent stimuli in SRT tasks (Mattes et al., 1997).

Nevertheless, when the results of the Posner task are discussed, the usual explanation for the observed differences between RT values for valid, neutral, and invalid cues is that the cue directs voluntary attention to the location it points to, which speeds up the processing of stimuli at that location. Part of the difference, however, might be due to the relative frequency effect. One may consider the relative frequency effect an attentional effect as well, wherein the most frequent stimuli capture attention because of their greater relevance. In any case, it is not because the cue indicates the most likely target location, but because the system was evolved, or trained, to respond faster when the cue is valid, as it could have been trained to respond faster when the cue is neutral or, for that matter, invalid. The effect of different cue ratios on the Posner task may be hard to detect, though, because in a noisy environment the attentional effect might be much stronger.

### 3.4. Noise

On the sensory stage of neuronal processing, noise may generate uncertainty about the presence of the cue and the target. The role of attention in filtering out distracting stimuli, so that they are excluded from decision and not confused with the target, has been extensively discussed in literature (Eriksen and Hoffman, 1973; Shiu and Pashler, 1994), and its synaptic mechanisms more recently probed (Briggs et al., 2013). In 1973, Eriksen and Hoffman observed that the presence of distracting stimuli close to the target increases RT (Eriksen and Hoffman, 1973), and later, Dosher and Lu (2000a) and Lu et al. (2002) performed discrimination experiments wherein attention, manipulated by the presence of valid and invalid cues, only influenced the results significantly in the presence of external noise. The mathematical model proposed by Reynolds and Heeger (2009) shows how a wide variety of proposed effects and attentional mechanisms can result from the same process through which the nervous system increases the sensitivity to signal and reduces the impact of noise.

In Condition C of Experiment 2, however, a strong effect of different cue types on RT was observed in the CRT task, but not in the SRT task, which indicates that the cue did not help to distinguish signal from noise. This might be because the ratio between signal intensity—the intensity of the target stimulus—and noise intensity—its standard deviation—was high, so the signal was clear. Such conclusion is also supported by the results of Experiment 1, wherein, at this signal-to-noise ratio, the Bayesian system detected the target within a short time from its onset, with little influence of the cue. When smaller signal-to-noise ratios were used instead (results not shown), the qualitative pattern of the results did not change, but the model's performance degraded, with error rates approaching 50% whenever the signal-to-noise ratio approached 0.5, and the networks took longer than *t*_*max*_ to respond, so that by that time the target had already appeared and RTs in SRT tasks did not depend on signal detection anymore.

An important difference between the neural network model and the Bayesian model is, though, that the former has only internal noise and the latter, only external noise. We may conclude that if external noise does not need to be filtered out, spatial cueing affects RT tasks more strongly when, in the motor stage of neuronal processing, the correct response must be selected; then the cue will be useful to inhibit the contralateral output neuron and to pre-activate the ipsilateral output neuron.

Brunton et al. (2013) have developed an evidence-accumulation model of perceptual decision-making that includes external and internal noise. By fitting the model's parameters to the results of perceptual tasks with rat and human subjects, they found that, for such tasks, the accumulator's memory is noiseless and only external noise is significant. Similarly, in our perceptual Bayesian model, only external noise is included; however, we have assumed that internal noise would still be relevant for motor tasks where response speed and accuracy matter, such as the Posner CRT task we studied.

It is our proposal that the cue is necessary for target detection in a noisy environment, but it affects motor output much more strongly when the task involves choosing an appropriate response than when there is only one response for all stimuli. This is also, to the best of our knowledge, a prediction that still has to be tested. There is only evidence that temporal expectancy, which we did not investigate, affects corticospinal excitability in a SRT task (van Elswijk et al., 2007), and no data from a CRT task for comparison.

### 3.5. Attention

In general, all results obtained from our experiments reflect the need to select adaptive actions, which generate reward. The relative frequency effect, for example, results from the simple strategy of optimizing for likely situations, which contribute more to the average performance than rare situations. In RT tasks, the moment a response occurs is crucial, since anticipated responses are not rewarded, and the best time to respond is soon after the target appears. Accordingly, when noise was introduced in the task, it became important to distinguish target from noise in low signal-to-noise ratio situations, as well as to slow down the activation of output neurons so that they would not fire in response to noise.

As already mentioned, when RT measurements are obtained from humans, the results are usually discussed with regard to attention. If we may also relate the results of our experiments to attention, then attention reflects the fact that some stimuli are more important than others to select an appropriate response. A common view of attention, known as “selection for action,” is that attention filters irrelevant stimuli so that only relevant stimuli will influence an individual's actions (Allport, 1987; Wu, 2011). In particular, Shiu and Pashler (1994) have proposed that, in RT tasks, the valid cue increases performance in noisy contexts, because perception is focused at the location pointed to by the cue and the processing of stimuli at other locations is inhibited, thus the probability that a distracting stimulus produce an incorrect response is decreased.

The standard view on selective attention, though, is that the nervous system can only deal with a limited amount of sensory information (Broadbent, 1958). When there is a stimulus overload, selection mechanisms are activated to ensure the processing of high priority stimuli (Desimone and Duncan, 1995). Mesulam stated that “If the brain had infinite capacity for information processing, there would be little need for attentional mechanisms” (Mesulam, 1985), and Posner also discussed his results from this perspective (Posner, 1980). In a 2011 review of visual attention research, attention has been defined as “a selective process, which is usually conceptualized as being related to limited cognitive and brain resources” (Carrasco, 2011). Indeed, the brain consumes energy in proportion to the number of neurons, and research indicates that each neuron has a fixed energy budget, which constrains its activity (Herculano-Houzel, 2011).

Limited capacity, however, does not explain our results. The filtering of noise and the control of the output circuits are necessary regardless of capacity. The Bayesian system, which exhibits a spatial “attentional effect,” i.e., a difference between RTs for the valid and the invalid cues, is not limited in any way. Rather, the effect results from different priors for different locations, which is required for optimal performance. Likewise, in a review by Dayan et al. (2000), a classical conditioning model implements selection by assigning different weights to stimuli in proportion to their reliability as predictors of a reward; selection mechanisms were necessary for good performance regardless of limited resources. The authors remarked that “selection is useful if the demands of a task imply that some available information is more relevant than other information (just as some available stimuli are more reliable predictors than others).” A similar argument was made by Krauzlis et al. (2014), who also proposed that attention arises from different weights being attributed to different inputs, which is required for value-based decision making.

In Chikkerur et al. (2010), spatial attention was also related to different spatial priors in a Bayesian model of perception, but it focused on a single object and location in order to reduce the complexity of learning probability distributions. But focusing on the target, as well as assigning different priors for different locations, are requirements for performing well in RT tasks and make it unnecessary to assume limited capacity.

Our results indicate that it is necessary to focus on what is relevant for action; the nervous system should inhibit distracting stimuli so that they won't influence decision making circuits and the most appropriate action can be selected based only on relevant stimuli. The capacity of the human nervous system is undoubtedly limited and the capacity of our neural networks is much more so, but our results do not follow from this limitation. The need to generate appropriate responses limits which stimuli may affect output circuits instead. In humans, an attentional “bottleneck” is the unitary nature of conscious process—to which attention is considered a necessary condition—and the inexorable fact that only one action must be selected and executed in a viable temporal window with ecologically advantageous results.

Our computational models have proved themselves useful to study visual attention through the fundamental problem of how to react fast and accurately to a stimulus. They have reproduced several results from RT experiments with human subjects: the relative frequency effect, suboptimal RTs and significant error rates due to noise and invalid cues, faster RTs for valid cues and slower RTs for invalid cues, slower RT for CRT tasks than for SRT tasks. More importantly, because our models are simple (in comparison to a living brain), it was possible to learn which mechanisms generated each result, what role such mechanisms played on the system's performance and how different factors affected them. Based on that learning, we have provided evidence for the validity of proposed mechanisms, as well as proposed novel mechanisms ourselves, which we hope will be experimentally supported.

## Author contributions

CF and MB created the models, CF programmed and ran the experiments, CF and MB wrote the paper.

### Conflict of interest statement

The authors declare that the research was conducted in the absence of any commercial or financial relationships that could be construed as a potential conflict of interest.

## Footnotes

^1^Even though neurons in Figure 5 have been drawn so that, for instance, the left cue neuron and the left output neuron are on the same side, they do not have actual positions in space. The model cannot exhibit stimulus-response compatibility effects, such as the Simon effect, either, because it has no structure prior to optimization that would make a given stimulus compatible or incompatible with a given response.
